# Association of MTHFR Polymorphisms with H-Type Hypertension: A Systemic Review and Network Meta-Analysis of Diagnostic Test Accuracy

**DOI:** 10.1155/2022/2861444

**Published:** 2022-03-22

**Authors:** Yixuan Kong, Jinghui Zheng, Lijuan Li, Liying Lu, Jie Wang

**Affiliations:** ^1^Ruikang Hospital Affiliated of Guangxi University of Chinese Medicine, Nanning 530011, Guangxi, China; ^2^Guangxi University of Chinese Medicine, Nanning 530011, Guangxi, China

## Abstract

**Purpose:**

An association between MTHFR polymorphisms and H-type hypertension (H-HTN) has been investigated by epidemiological studies, but results have been inconsistent. Thus, a systematic assessment of the association was performed based on a literature review and pooled analysis, to provide stronger evidence on the effects of single nucleotide polymorphisms on H-HTN risk.

**Methods:**

Three investigators independently retrieved relevant studies in PubMed, Embase, Cochrane Library, China National Knowledge Infrastructure (CNKI), China Science and Technology Journal Database (VIP), Wanfang Database, and China Biomedical Literature Database (CBM). A fixed or random effects model was selected to calculate pooled odds ratio (OR) and 95% confidence intervals (CIs). A network meta-analysis of diagnostic test and Thakkinstian's algorithm were used to select the most appropriate genetic model, along with false-positive report probability (FPRP) for noteworthy associations. All data were processed using Stata 15.0 and Meta-Disc.

**Results:**

A total of 14 studies involving 1759 cases and 1581 controls for MTHFR were included in our meta-analysis. In a direct meta-analysis, we found that MTHFR C667T rs1801133 significantly increased the risk of H-HTN susceptibility except for an overdominant model. However, MTHFR A1298C rs1801131 polymorphism had no significant correlation with H-HTN risk. Besides, MTHFR C667T rs1801133 is a potential diagnostic biomarker for estimating H-HTN risk. The results indicated that the dominant model was an optimal diagnosis model for excluding diseases, which could reduce a missed diagnosis rate and further improve the accuracy of disease diagnosis.

**Conclusion:**

The present result suggests that MTHFR C667T rs1801133 polymorphism is associated with H-HTN risk and may act as a promising predictive biomarker for H-HTN risk. However, further well-designed studies are warranted to confirm these results.

## 1. Introduction

H-type hypertension (H-HTN) is a form of essential hypertension (HTN) often associated with hyperhomocysteinemia (HHCY) [1]. When human blood homocysteine level is above 15 *μ*mol/l, it is called hyperhomocysteinemia, and essential HTN accompanied by this sign is defined as H-HTN [2]. H-HTN accounts for 75% of HTN cases in China [3]. However, there is limited research on H-HTN in the Western countries, which needs attention. H-HTN is caused by many factors, including age, gender, body mass index, sodium intake, uric acid level, creatinine level, and low-density lipoprotein cholesterol level [4]. However, environmental and genetic factors and their interactions have an important impact on the pathogenesis of H-HTN [5]. Evidence indicated that between 30 and 60% of the variability associated with blood pressure is inherited [6]. Meanwhile, genetic factors may result in abnormal homocysteine. Among genetic determinants of homocysteinemia, the C677T polymorphism of methylenetetrahydrofolate reductase (MTHFR) gene is the most important and most studied [7–9]. According to research, MTHFR plays a major role in regulating homocysteine (HCY) levels. HCY decreases dramatically with the C677T mutation in the gene [10]. As a key enzyme in the metabolism of homocysteine, MTHFR converts 5,10-methylenetetrahydrofolate into 5-methyltetrahydrofolate, and this reaction provides a methyl for homocysteine form methionine [11, 12]. C677T polymorphisms were assessed as potential candidate genes of MTHFR [13]. The C677T polymorphism of the MTHFR gene has been identified as the most important genetic risk factor for elevated homocysteine, substitution of cytosine (C) by thymine (T), which converts an alanine to a valine residue. C677T mutation results in a significantly decreased activity, which eventually leads to an elevated plasma HCY concentration [14, 15].

Previously, some epidemiological studies have investigated the relationship of the MTHFR gene with H-HTN; for example, a study showed that MTHFR C677T mutation might predict essential HTN in the Spanish male population [16]. However, other studies have come to the opposite conclusion [17]. Due to inconsistent results, this meta-analysis based on our research protocol registered in the INPLASY will provide stronger evidence on the effects of single nucleotide polymorphisms (SNPs) on H-HTN risk [18–31]. We screened suitable genetic models and used a false-positive report probability (FPRP) to determine the most strongly associated SNPs with H-HTN susceptibility. Then, using the network meta-analysis method to evaluate and compare the diagnostic accuracy of genetic models and rank these models, we found the best model to diagnose H-HTN.

## 2. Material and Methods

This study was conducted in accordance with the PRISMA (Preferred Reporting Items for Systematic Reviews and Meta-Analyses) guidelines, and the protocol was registered in the INPLASY (INPLASY202050002).

### 2.1. Identification of Eligible Studies

We systematically searched in PubMed, Embase, Cochrane Library, China National Knowledge Infrastructure (CNKI), China Science and Technology Journal Database (VIP), Wanfang Database, and Chinese Biomedical Literature Database (CBM) up to June 2021. The following search terms were used: single nucleotide polymorphism, SNP, homocysteine, and H-type hypertension.

### 2.2. Inclusion Criteria

Studies in this meta-analysis met the following inclusion criteria:(1) studied patients with a definite diagnosis of H-HTN; (2) studied single nucleotide polymorphisms; (3)compared to non-H-HTN; (4) used case-control design; (5) used homocysteine ≥15 umol/L as a reference standard; (6) had no restrictions on age, gender, and country; (7) included only the largest study when there were multiple same research publications from the same research team.

### 2.3. Exclusion Criteria

Exclusion criteria were (1) duplication report, conference report, thesis, review paper, animal study, or poster; (2) studies without detailed genotypic distribution data; (3) studies in which SNPs demonstrated a departure from Hardy–Weinberg equilibrium (HWE; *p* < 0.05) in controls; and (4) non-Chinese or English literature.

### 2.4. Data Extraction

Qualified data were extracted from the included studies by two investigators, and any disagreement was resolved by a third investigator to reach a consensus on this (Supplementary Figure 1 is the PRISMA flow diagram illustrating the procedure of study selection). The following data from studies were selected: first author's name, publication year, methods of gene detection, country, region, race, number of cases and controls, genotype frequencies, and HWE values. If original data were unavailable, we contacted a corresponding author.

### 2.5. Qualitative Evaluation

The qualities of the included studies were accessed based on the STREGA statement [32] by two investigators according to the methodological quality assessment, and the third investigator resolved any disagreement. Evaluation criteria included the following items: (1) whether the exposed genes were clearly defined; (2) whether the genotyping method was described; (3) whether HWE was considered in the control group; (4) whether the genotype was inferred; (5) whether the population stratification method was described; (6) whether the diagnostic criteria were clear; (7) whether the data were sufficient; and (8) whether statistical methods and software were described (Supplementary Table 1).

### 2.6. Statistical Analysis

In the included studies, 3 SNPs were associated with the risk of H-HTN. However, only one study of ALDH2 rs671 could not be conducted by meta-analysis. The remaining two studies used the Stata software 15.1 for data processing and analyzed six genetic models: allele contrast model, homozygous model, heterozygous model, dominant model, recessive model, and overdominant model. We calculated pooled odds ratio (OR) and 95% confidence intervals (CIs) for pairwise meta-analysis. Heterogeneity among the models was quantified with the I^2^ statistics and *P* value: I^2^ statistics <50% and *P* > 0.1 indicated low heterogeneity. When high heterogeneity existed, meta-regression was performed to detect a source of heterogeneity, and subgroup analysis was conducted. If the source of heterogeneity could not be found, a random effects model was employed; otherwise, a fixed effects model was adopted. Sensitivity analysis was conducted to check each study robustness and reliability of pooled results, and the publication bias was assessed using Begg's and Egger's tests.

Furthermore, we selected the most appropriate model adopted by Thakkinstian et al.'s algorithm [33]. An SNP consists of dominant (*G*) and recessive alleles (g). In the paired meta-analysis, we calculated the pool OR values: OR1, OR2, and OR3 for GG versus gg (D1), Gg versus gg (D2), and GG versus Gg (D3), along with 95% CIs. Then, we selected the most appropriate genetic model: if OR1 = OR3 = 1 and OR2 = 1, a recessive model was suggested; if OR1 = OR2 = 1and OR3 = 1, a dominant model was suggested; if OR2 = 1 = OR3 = 1 and OR1 = 1, a complete overdominant model was used; and if OR1 > OR2 > 1 and OR1 > OR3 > 1 or OR1 < OR2 < 1, and OR1 < OR3 < 1, a codominant model was used. We could not judge whether these genetic models had a real correlation with H-HTN, so we used the false-positive report probability (FPRP) [34] to determine this. The default FPRP threshold was 0.2, by assuming a three-level prior probability (high: 0.1; moderate: 0.01; low: 0.001). The OR value was set to 1.5. When the FPRP value was less than 0.2, the probability of false positive is low.

To determine the diagnostic value of different genetic models for H-HTN, we calculated TP, FP, FN, TN, and FN values by converting the number of exposed and nonexposed genes in the SNP model into a diagnostic test. Diagnostic meta-analysis was conducted by using the Meta-Disc software and evaluated the sensitivity, specificity, likelihood ratios (LRs), diagnostic advantage ratios (DORs), and summary receiver-operating characteristic curve (SROC). An evaluation of heterogeneity related to threshold effect by Spearman correlation analysis.

We compared the data with the common gold standard and performed a network meta-analysis of diagnostic test accuracy studies. When the *P* value was <0.5, obvious deviation existed, so we adopted the inconsistent model. If there was heterogeneity, the random effects model was used to compare its sensitivity and specificity with the gold standard. The results were evaluated using the pooled risk ratio (RR) and 95% CI. We ranked the probability of each genetic model for risk assessment and generated corresponding ranking probability maps.

## 3. Results

### 3.1. Characteristics of Eligible Studies

A total of 228 studies were obtained from PubMed, Embase, Cochrane, CNKI, Wanfang Database, China Science and Technology Journal Database (VIP), and China Biomedical Literature Database (CBM). The PRISMA flow chart summarizes this literature selection process. The search yielded 228 studies on SNPs associated with H-HTN. After screening out duplicate records and reading titles and abstracts, 183 unrelated studies were excluded. After reading full texts, 31 studies were excluded. Finally, 14 qualified studies were included involving 1594 cases and 1431 controls for MTHFR C667T rs1801133 and 165 cases and 150 controls for MTHFR A1298C rs1801131, which were analyzed in our meta-analysis. Different genotyping methods were employed, including polymerase chain reaction (PCR), polymerase chain reaction-restriction fragment length polymorphism (PCR-RFLP), and allele specific-polymerase chain reaction (AS-PCR). Control groups in all studies were included in accordance with HWE.

Supplementary Table 2 presents the main characteristics of included studies and the results of the HWE test.

### 3.2. Pairwise Meta-Analysis

Since only one study investigated ALDH2 rs671, we were unable to perform a related meta-analysis. Therefore, the other two SNPs were investigated for an association with H-HTN risk in this study. MTHFR C667T rs1801133 polymorphism significantly increased the risk of H-HTN under all genetic models except for the overdominant model (C versus T: OR = 2.17, 95% CI 1.77–2.66, I^2^ = 71.2%; CC versus TT: OR = 4.36, 95% CI 2.74–6.96, I^2^ = 70.6%; CC versus CT: OR = 1.59, 95% CI 1.28–1.96, I^2^ = 33.7%; CT/CC versus TT: OR = 2.19, 95% CI 1.76–2.73, I^2^ = 46.5%; CC versus TT/CT: OR = 3.23, 95% CI 2.14–4.87, I^2^ = 71.4%; CC/TT versus CT: OR = 1.08, 95% CI 0.85–1.36, I^2^ = 57.2%), as shown in Supplementary Figure 2. for MTHFR A1298C rs1801131, we observed the trend of risk reduction, and no significant association was found in any genetic model (A versus C: OR = 1.02, 95% CI 0.7–1.5, I^2^ = 0.00%; AA versus CC: OR = 0.95, 95% CI 0.47–2.13, I^2^ = 0.00%; AC versus CC: OR = 1.09, 95% CI 0.65–1.83, I^2^ = 0.00%; AC/CC versus AA: OR = 1.06, 95% CI 0.66–1.69, I^2^ = 0.00%; CC versus AA/AC: OR = 0.94, 95% CI 0.47–2.08, I^2^ = 0.00%; CC/AA versus AC: OR = 0.91, 95% CI 0.55–1.52, I^2^ = 0.00%), as shown in Supplementary Figure 3.

Through I^2^ statistics and *P* values, we found that heterogeneity of MTHFR C667T rs1801133 exists under allele contrast, homozygous, recessive, and overdominant models. A meta-regression detected the source of heterogeneity. Meta-regression results showed that these factors do not explain heterogeneity between studies (*P* > 0.05). Thus, we used the random effects model for the effect sizes.

Sensitivity analysis was performed to explore the influence of individual studies on the pooled results for MTHFR C667T rs1801133 by deleting each study every time from the pooled analysis. The results showed that heterogeneity remained unchanged, indicating that the pooled outcome results were relatively robust. Due to a limited number of studies (*n* < 10), A1298C rs1801131 was no longer included in the analysis.

### 3.3. Evaluation of Publication Bias

Publication bias was examined by Begg's funnel plot and Egger's test. The calculation results indicated that Begg's funnel plot and Egger's test of all models had *P* > 0.05, except for the homozygous model (Begg's test *P* = 0.003, Egger's test *P* = 0.01) and recessive model (Begg's test *P* = 0.014, Egger's test *P* = 0.03), which were considered as publication bias (Supplementary Figure 4).

### 3.4. Determination of the Most Appropriate Genetic Models

We selected the most appropriate genetic model using Thakkinstian's algorithm. For *MTHFR* C667T rs1801133, we calculated OR1, OR2, and OR3 separately: 0.23 (0.14, 0.37), 0.38 (0.25, 0.58), and 0.62 (0.51, 0.77). The results suggested that the genetic model was most likely to be codominant.

The FPRP was calculated in significant SNPs related to H-HTN risk (Supplementary Table 3). When a prior probability was 0.001 and OR was 1.5, for *MTHFR* C667T rs1801133, the overdominant model FPRP value higher than 0.2, indicated that the significant correlation of this genetic model with H-HTN risk was false positive. Another five risk models' FPRP values were all below 0.2; thus, these models were noteworthy for increasing the risk of H-HTN.

### 3.5. Meta-Analysis of Diagnostic Test

A meta-analysis of diagnostic tests was performed to determine the performance of *MTHFR* C667T rs1801133 for identifying H-HTN. We selected dominant, recessive, and overdominant genetic models containing three genotypes. Taking DOR as effect quantity, we analyzed heterogeneity of three genetic models (I^2^ = 42.8%, I^2^ = 70.8%, I^2^ = 72.7%). In both recessive and overdominant genetic models, Spearman correlation coefficients were 0.63 (*P* = 0.05) and 0.441 (*P* = 0.067), respectively, suggesting that the heterogeneity might be caused by threshold effect, and the random effects model was adopted to merge. For the dominant model, pooled DOR, sensitivity, specificity, +LR, and −LR were 2.16 (95% CI, 1.76–2.65), 0.69 (95% CI, 0.67–0.72), 0.48 (95% CI, 0.46–0.51), 1.31 (95% CI, 1.21–1.42), and 0.64 (95% CI, 0.56–0.73), respectively, and the SROC curve was 0.612. For the recessive model, the summary DOR, sensitivity, specificity, +LR, and −LR were 3.08 (95% CI, 2.1–4.5), 0.37 (95% CI, 0.35–0.39), 0.83 (95% CI, 0.82–0.85), +LR was 1.99 (95% CI, 1.57–2.52), and −LR was 0.77 (95% CI, 0.7–0.83), respectively, and the SROC curve was 0.607. For the overdominant model, the summary DOR, sensitivity, specificity, +LR, and −LR were 1.21 (95% CI, 0.91–1.61), 0.61 (95% CI, 0.58–0.63), 0.44 (95% CI, 0.42–0.47), 1.10 (95% CI, 0.99–1.23), and 0.87 (95% CI, 0.74–1.02), respectively, and the SROC curve was 0.525. The results suggested that the three genetic models of *MTHFR* C667T rs1801133 have a certain accuracy as diagnostic indicators for H-HTN. However, the accuracy was low and needs to be further tested (SROC curve is shown in Supplementary Figure 5).

### 3.6. Network Meta-Analysis of Diagnostic Test

Then, the sensitivity and specificity of the abovementioned models were compared with the common gold standard (HCY ≥15 *μ*mol/L). Under the inconsistent model, the indirect comparison among the three was formed, thereby for a network (Supplementary Figure 6). In terms of sensitivity, the dominant model (RR = 1.41, 95% CI 1.22–1.64) was superior to other genetic models (Supplementary Figure 7), and a ranking probability map showed that H-HTN risk estimation was from best to worst: dominant > over dominant > recessive. In terms of specificity, the pooled RR and CI for the dominant model, recessive model, and overdominant model were 2.14, 1.88–2.45; 0.96, 0.83–1.11; and 1.78, 1.55–2.03; respectively (Supplementary Figure 7). The results suggested that the recessive model had no statistical significance in reducing the misdiagnosis rate of H-HTN (Rank probability shown in supplementary Figure 8).

## 4. Discussion

Herein, we found that elevated H-HTN was associated with *ALDH2* rs671 and *MTHFR* C677T rs1801133 and A1298C rs1801131. Due to a limited number of studies, we were unable to conduct a meta-analysis for *ALDH2*; hence, this meta-analysis focused on *MTHFR* gene polymorphisms association with the risk of H-HTN.

HHCY is associated with H-HTN, and its regulatory gene MTHFR has become a new biomarker of H-HTN [35]. MTHFR gene is located at p36.3 on human chromosome no.1. So far, more than 40 MTHFR gene point mutations have been identified. For C677T, the substitution of C-nucleotides by T-nucleotides leads to the change of amino acids from alanine to valine. This genetic mutation results in reduced MTHFR activity, elevated plasma homocysteine, and altered folate distribution, with MTHFR activity in carriers of the TT and CT genotypes being 70% and 35% lower than in normal subjects [36, 37], respectively. Kang et al. [38] performed a meta-analysis in 2013 and found that the C677T mutation was associated with a high risk for hemorrhagic stroke, especially the *T* allele. Also, for the A/C variant at site 1298, which converts its encoded glutamic acid into alanine, we found that this SNP had no significant association with H-HTN. However, some studies have shown that this mutation impacts the increase in HCY level [39, 40]. There are inconsistencies in results that may be related to the subjects' race, that should be treated with caution.

Previously, Wu et al. [41] performed a meta-analysis of 30 studies, which supported a role for MTHFR C677T polymorphism in development of hypertension. A recent meta-analysis showed that MTHFR C677T gene polymorphism was associated with an increased risk of hypertension in the overall population, East Asian and Caucasian populations. The above association became less reliable after FPRP, BFDP, and Venice criteria tests. False positives may exist and should be further verified [42]. Both studies showed no significant association between MTHFR A1298C polymorphism and hypertension [41, 42]. However, those meta-analyses only studied essential hypertension and did not focus on the association between MTHFR polymorphisms and the susceptibility to H- type hypertension.

According to our search of the existing public database, we found that there was no study on the network meta-analysis of diagnostic tests to evaluate and compare the accuracy of SNP models for H-HTN. This study screened out the case group confirmed as H-HTN, through each gene model compared to the gold standard (HCY ≧15 *μ*mol/l), with the gold standard serving as a reference, thereby forming an indirect comparison among the models. The relationship between the MTHFR gene and H-HTN risk was analyzed from the two aspects of etiology and diagnosis of H-HTN.

In the direct meta-analysis, we analyzed 6 gene models of SNPs of C677T rs1801133 and A1298C rs1801131. C677T rs1801133 was significantly associated with H-HTN risk, while A1298C was associated with reduced risk. In C677T, except for the overdominant model, which had no significant correlation with H-HTN risk, the other models correlated with H-HTN risk. However, in the diagnostic meta-analysis, the MTHFR C677T predicted the risk of H-HTN for approximately 50%–60% that is in the low position of sensitivity and specificity. Therefore, MTHFR C677T cannot be used alone for the diagnosis of H-HTN among high-risk populations but can be used as an auxiliary diagnostic tool together with other screening methods to improve the effectiveness of screening for H-HTN. In the network diagnosis meta-analysis, the dominant model, recessive model, and overdominant model with three genotypes were used as diagnostic indicators, and their sensitivity and specificity were analyzed in combination with the gold standard. The results of probability ranking showed that the dominant model was the optimal diagnosis model for excluding H-HTN, which could reduce the missed diagnosis rate and further improve the accuracy of diagnosis. The patients with negative results were excluded. The second is recessive model. In the diagnosis of H-HTN, the dominant model is also the best diagnostic model, and its high specificity indicates that the misdiagnosis rate is low, and the overdominant model has high accuracy in the diagnosis of H-HTN.

The prevalence of MTHFR 677 CT and TT genotypes was much higher in this Chinese population than previously reported in other populations. We found that 27% had CC, 49% had CT, and 24% had the TT genotype [43]. Yang et al. [44] reported a Chinese population prevalence for HCY >15 µmol/L of 27.5%. This may result in a higher prevalence of H-HTN in China than in other populations.

This study has several strengths. In contrast to most meta-analyses on hypertension, this is the first meta-analysis focused on the association between MTHFR polymorphisms and the susceptibility to H-type hypertension. Secondly, we innovated to use network meta-analysis to compare the diagnostic accuracy of different gene models for the same gene combined with the gold standard in H-HTN diagnosis and rank the diagnostic effects. False-negative results can lead to a missed diagnosis of H-HTN and delay treatment of patients. False-positive results will lead to misdiagnosis and increase unnecessary medical expenses. This study indicated that the MTHFR gene is an important biomarker for H-HTN and can assist in the diagnosis of H-HTN, allowing timely treatment, and changing treatment strategies in time by clinicians. Thirdly, Thakkinstian et al.'s algorithm was used select the most appropriate model, and the authenticity of the meta-analysis was also measured by the FPRP to confirm that the gene is closely related to H-HTN susceptibility. In addition, all included studies had high quality assessment scores.

Our study has several limitations. Firstly, the sample size of this study is small because we included only patients with a clear diagnosis of H-HTN. Secondly, the subjects of the study are all Chinese people, and thus, there are ethnic limitations. Thirdly, the heterogeneity of the genetic models might be caused by different studies.

## 5. Conclusion

In conclusion, A1298C rs1801131 is not associated with the susceptibility to H-HTN, while *MTHFR* C677T has a significant association with HTN risk and is an ideal biomarker for the diagnosis of H-HTN. The dominant model is the appropriate model for detecting the risk association with H-HTN and predicting H-HTN. However, further verification of the role of the *MTHFR* gene in H-HTN requires a larger sample size and more ethnicity.

## Abbreviations:

HCY: Homocysteine
SNPs: Single nucleotide polymorphisms
H-HTN: H-type hypertension
MTHFR: Methylenetetrahydrofolate reductase
ALDH2: Acetaldehyde dehydrogenase 2
PRISMA: Preferred Reporting Items for Systematic Reviews and Meta-Analyses
HWE: Hardy–Weinberg equilibrium
CNKI: China National Knowledge Infrastructure
VIP: Chinese Science and Technology Periodical Database
CBM: Chinese Biomedical Literature Database
STREGA: Strengthening the Reporting of Genetic Association Studies
CIs: Confidence intervals
OR: Odds ratio;
RR: Risk ratio
FPRP: False-positive report probability
SROC: Summary receiver-operating characteristic
AUC: Area under the curve
+LR: Positive likelihood ratio
−LR: Negative likelihood ratio
DOR: Diagnostic odds ratio.

## Supplementary Materials

Supplementary Materials Supplementary Figure 1: PRISMA flow diagram of literature search and selection. Supplementary Figure 2: forest plot of MTHFR C667T rs1801133. Supplementary Figure 3: Forest plot of MTHFR A1298C rs1801131. Supplementary Figure 4: Begg's funnel plot and Egger's test. Supplementary Figure 5: SROC curve. Supplementary Figure 6: network evidence plot for H-hypertension. Supplementary Figure 7: risk ratio and 95% CI. Supplementary Figure 8: rank probability. Supplementary Table 1: quality evaluation results. Supplementary Table 2: characteristics of studies included in the meta-analysis. Supplementary Table 3: false-positive report probability (FPRP) for selected genetic model.
