# Supplementary material for: Association of MTHFR Polymorphisms with H-Type Hypertension: A Systemic Review and Network Meta-Analysis of Diagnostic Test Accuracy
Source: Int J Hypertens. 2022 Mar 22;2022:2861444. doi: 10.1155/2022/2861444 (PMC8964216; doi:10.1155/2022/2861444)
Supplement: Supplementary Materials — Supplementary Figure 1: PRISMA flow diagram of literature search and selection. Supplementary Figure 2: forest plot of MTHFR C667T rs1801133. Supplementary Figure 3: Forest plot of MTHFR A1298C rs1801131. Supplementary Figure 4: Begg's funnel plot and Egger's test. Supplementary Figure 5: SROC curve. Supplementary Figure 6: network evidence plot for H-hypertension. Supplementary Figure 7: risk ratio and 95% CI. Supplementary Figure 8: rank probability. Supplementary Table 1: quality evaluation results. Supplementary Table 2: characteristics of studies included in the meta-analysis. Supplementary Table 3: false-positive report probability (FPRP) for selected genetic model. [file 2861444.f1.docx]

**Supplementary Figure 1. PRISMA flow diagram of literature search and selection**

Duplicate records

(n=145)

Additional records identified through other sources
(n = 0 )

228 of records identified through databases searching

(PubMed(n=1) ,Embase(n=1),

Cochrance(n=12) CNKI(n=54),WanFang n=72）VIP(n=41),CBM(n=47)

(n =228)

Full-text articles excluded,

Non-case control study

(n =5 )

Combined with other diseases studies(n=7)

Fail to find out full article

(n=2)
letter, Conference reports, overview, Research progress (n=7)

lack of allele frequencies

(n=4)

Irrelevant research(n=6)

Records excluded

After reading abstract and title
(n =38)

Full-text articles assessed for eligibility
(n =14 )

Records screened
(n =45 )

Records after duplicates removed
(n =83)

**Supplementary Figure.2 Forest plot of MTHFRC667T rs1801133**

**Supplementary Figure.3 Forest plot of MTHFR A1298C rs1801131**

**Supplementary Figure 4. Begg’s funnel plot and Egger’s test**


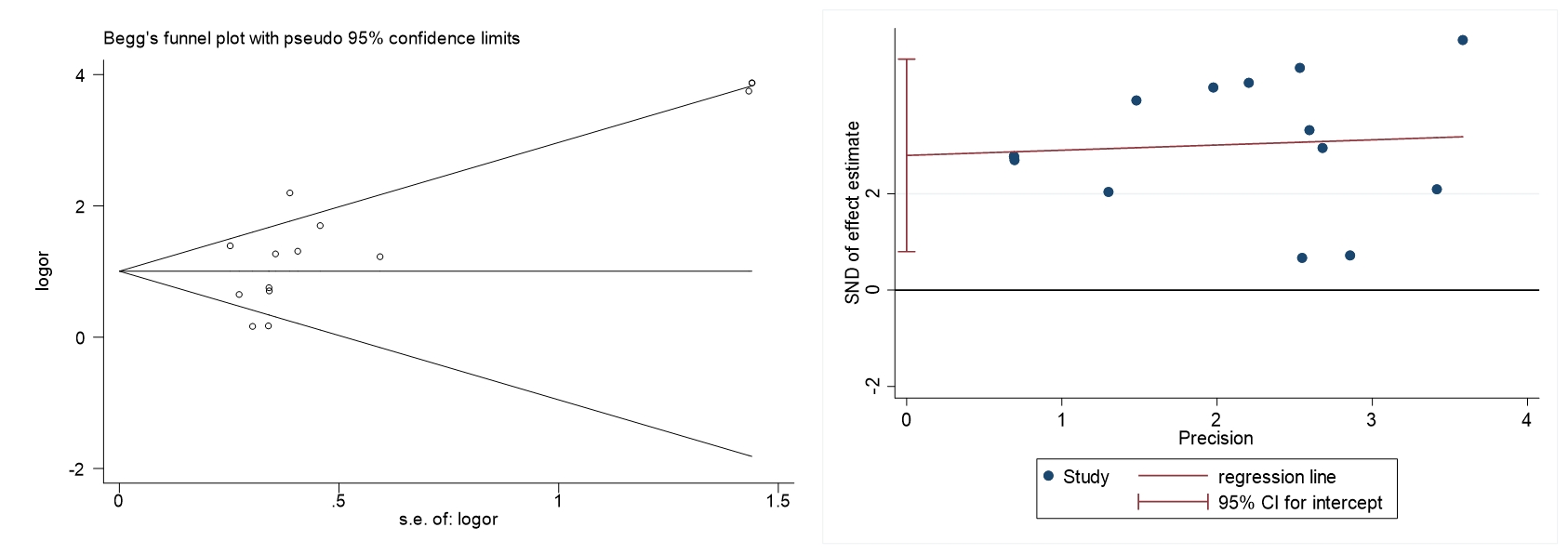


**Supplementary Figure 5. SROC curve**


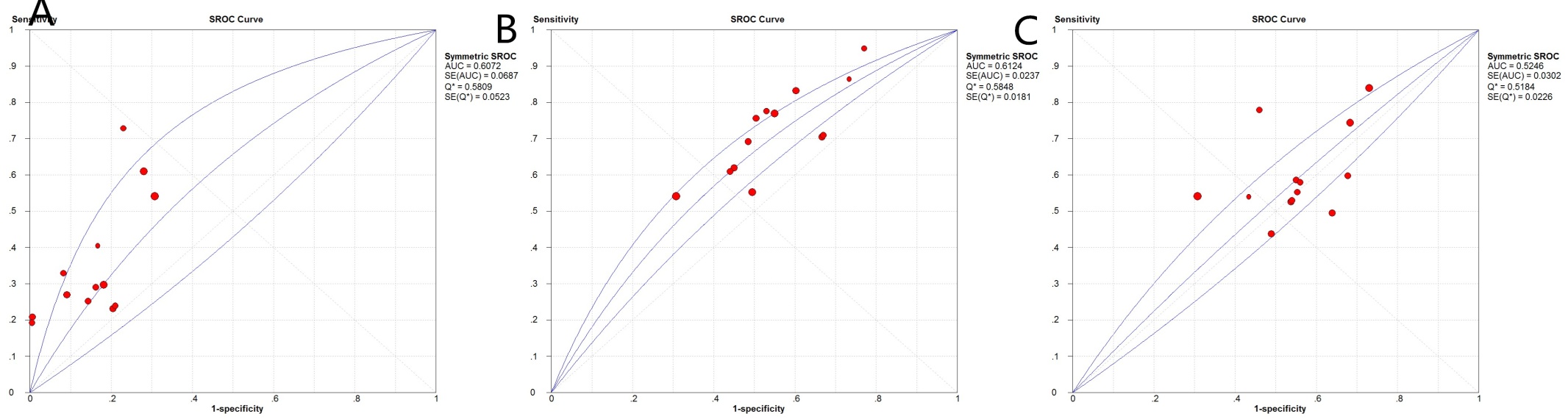


**NOTE**:A=dominant model. B=recessive model. C=over-dominant model

**Supplementary Figure 6. Network evidence plot for H-hypertension**


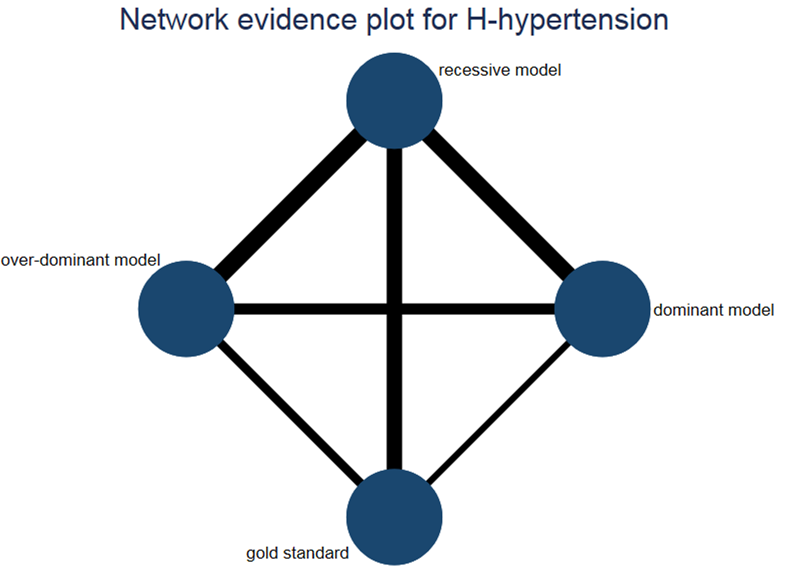


**Supplementary Figure 7. risk ratio and 95%CI**

| A | 1.69(1.45,1.97) | 3.01(2.55,3.56) | 1.41（1.22,1.64） |
| --- | --- | --- | --- |
| 2.24(1.96,2.56) | B | 1.78（1.50,2.11） | 0.84（0.72,0.98） |
| 1.21(1.07,1.36) | 0.54(0.47,0.62) | C | 0.47(0.40,0.56) |
| 2.14(1.88,2.45) | 0.96(0.83,1.11) | 1.78(1.55,2.03) | D |
| **NOTE：**Sensitivity（pper right），Specificity（Left below）；A= dominant model，B= recessive model, C= over dominant model, D= gold standard | | | |

**Supplementary Figure 8. Rank probability**

| **Supplementary Table 1 The quality evaluation results** | | | | | | | | | |
| --- | --- | --- | --- | --- | --- | --- | --- | --- | --- |
| Study id | ① | ② | ③ | ④ | ⑤ | ⑥ | ⑦ | ⑧ | Score |
| Zhengyu Cao | 1 | 1 | 1 | 0 | 0 | 1 | 1 | 1 | 6 |
| Chunfang LV | 1 | 1 | 1 | 0 | 0 | 1 | 1 | 1 | 6 |
| Shaoyan Zhu | 1 | 1 | 1 | 0 | 0 | 1 | 1 | 1 | 6 |
| Jia Wang | 1 | 1 | 1 | 0 | 1 | 1 | 1 | 1 | 7 |
| Lei Chen | 1 | 1 | 1 | 0 | 1 | 1 | 1 | 1 | 7 |
| Congna Li | 1 | 1 | 1 | 0 | 0 | 1 | 1 | 1 | 6 |
| Zhi Tang | 1 | 1 | 1 | 0 | 0 | 1 | 1 | 1 | 6 |
| Yingying Zhang | 1 | 1 | 1 | 0 | 0 | 1 | 1 | 1 | 6 |
| Fang An | 1 | 1 | 1 | 0 | 1 | 1 | 1 | 1 | 7 |
| Jing Qian | 1 | 1 | 1 | 0 | 1 | 1 | 1 | 1 | 7 |
| Hanshu Zhang | 1 | 1 | 1 | 0 | 0 | 1 | 1 | 1 | 6 |
| Suyan Bian | 1 | 1 | 1 | 0 | 0 | 1 | 1 | 1 | 6 |
| Xiaoye Su | 1 | 1 | 1 | 0 | 0 | 1 | 1 | 1 | 6 |
| Xiaohui Lin | 1 | 1 | 1 | 0 | 0 | 1 | 1 | 1 | 6 |
| **Note:**①Whether the exposed genes were clearly defined; ② whether the genotyping method was described;③whether HWE was considered in the control group; ④ whether the genotype was inferred; ⑤whether the population stratification method was described; ⑥ whether the diagnostic criteria were clear; ⑦ whether the data were sufficient; ⑧whether the statistical methods and software were described | | | | | | | | | |

| \| **Supplementary Table 2** Characteristics of studies included in the meta-analysis \| \| \| \| \| \| \| \| \| \| \| \| --- \| --- \| --- \| --- \| --- \| --- \| --- \| --- \| --- \| --- \| --- \| \| Study id \| Year \| Country \| Detection method \| Case \|  \|  \| Control \|  \|  \| P for HWE \| \| MTHFR C677T rs1801133 \| \|  \|  \| TT \| TC \| CC \| TT \| TC \| CC \|  \| \| Zhengyu Cao \| 2012 \| China \| PCR \| 26 \| 53 \| 33 \| 30 \| 68 \| 49 \| 0.767 \| \| Chunfang LV \| 2014 \| China \| PCR-RFLP \| 31 \| 62 \| 57 \| 0 \| 36 \| 44 \| 0.45 \| \| Shaoyan Zhu \| 2015 \| China \| PCR-RFLP \| 24 \| 47 \| 29 \| 21 \| 46 \| 33 \| 0.568 \| \| Jia Wang \| 2015 \| China \| PCR-RFLP \| 28 \| 24 \| 42 \| 52 \| 91 \| 144 \| 0.141 \| \| Lei Chen \| 2016 \| China \| PCR-RFLP \| 15 \| 17 \| 5 \| 5 \| 17 \| 8 \| 0.726 \| \| Congna Li \| 2016 \| China \| PCR \| 44 \| 68 \| 32 \| 12 \| 43 \| 54 \| 0.443 \| \| Zhi Tang \| 2017 \| China \| PCR-RFLP \| 19 \| 42 \| 39 \| 0 \| 44 \| 56 \| 0.536 \| \| Yingying Zhang \| 2017 \| China \| PCR-RFLP \| 50 \| 104 \| 31 \| 8 \| 45 \| 35 \| 0.22 \| \| Fang An \| 2018 \| China \| PCR-RFLP \| 34 \| 47 \| 36 \| 16 \| 32 \| 51 \| 0．251 \| \| Jing Qian \| 2018 \| China \| AS-PCR \| 28 \| 56 \| 27 \| 17 \| 43 \| 59 \| 0.1 \| \| Hanshu Zhang \| 2019 \| China \| PCR \| 28 \| 38 \| 19 \| 7 \| 38 \| 40 \| 0.626 \| \| Suyan Bian \| 2019 \| China \| PCR \| 43 \| 13 \| 3 \| 20 \| 47 \| 20 \| 0.756 \| \| Xiaoye Su \| 2019 \| China \| PCR-RFLP \| 183 \| 48 \| 69 \| 28 \| 27 \| 45 \| 0.13 \| \|  \|  \|  \|  \|  \|  \|  \|  \|  \|  \|  \| \| MTHFR A1298C rs1801131 \| \| \|  \| AA \| AC \| CC \| AA \| AC \| CC \|  \| \| Xiaohui Lin \| 2012 \| China \| PCR-RFLP \| 54 \| 17 \| 9 \| 42 \| 15 \| 8 \| 0.82 \| \| Hanshu Zhang \| 2019 \| China \| PCR \| 54 \| 26 \| 5 \| 58 \| 22 \| 5 \| 0.158 \| |  |
| --- | --- | --- | --- | --- | --- | --- | --- | --- | --- | --- | --- | --- | --- | --- | --- | --- | --- | --- | --- | --- | --- | --- | --- | --- | --- | --- | --- | --- | --- | --- | --- | --- | --- | --- | --- | --- | --- | --- | --- | --- | --- | --- | --- | --- | --- | --- | --- | --- | --- | --- | --- | --- | --- | --- | --- | --- | --- | --- | --- | --- | --- | --- | --- | --- | --- | --- | --- | --- | --- | --- | --- | --- | --- | --- | --- | --- | --- | --- | --- | --- | --- | --- | --- | --- | --- | --- | --- | --- | --- | --- | --- | --- | --- | --- | --- | --- | --- | --- | --- | --- | --- | --- | --- | --- | --- | --- | --- | --- | --- | --- | --- | --- | --- | --- | --- | --- | --- | --- | --- | --- | --- | --- | --- | --- | --- | --- | --- | --- | --- | --- | --- | --- | --- | --- | --- | --- | --- | --- | --- | --- | --- | --- | --- | --- | --- | --- | --- | --- | --- | --- | --- | --- | --- | --- | --- | --- | --- | --- | --- | --- | --- | --- | --- | --- | --- | --- | --- | --- | --- | --- | --- | --- | --- | --- | --- | --- | --- | --- | --- | --- | --- | --- | --- | --- | --- | --- | --- | --- | --- | --- | --- | --- | --- | --- | --- | --- | --- | --- | --- | --- | --- | --- | --- | --- | --- | --- | --- | --- | --- | --- | --- | --- | --- | --- | --- | --- | --- | --- | --- | --- | --- |

| **Supplementary Table3**  False positive report probability (FPRP) for selected genetic model | | |
| --- | --- | --- |
| Genetic model | OR(95%CI) | FPRP prior probability |
| allele contrast model | 2.17(1.77-2.66) | 0.00 |
| homozygous model | 4.36(2.74-6.96) | 0.149 |
| heterozygous model | 1.59(1.28-1.96) | 0.045 |
| dominant model | 2.19(1.76-2.73) | 0.00 |
| recessive model | 3.23(2.14-4.87) | 0.148 |
| over-dominant model | 1.08(0.85-1.36) | 0.998 |
